# The Autophagy Level Is Increased in the Synovial Tissues of Patients with Active Rheumatoid Arthritis and Is Correlated with Disease Severity

**DOI:** 10.1155/2017/7623145

**Published:** 2017-02-01

**Authors:** Li Zhu, Huaizhou Wang, Yu Wu, Zhengwen He, Yanghua Qin, Qian Shen

**Affiliations:** ^1^Department of Laboratory Medicine, Affiliated Wuxi People's Hospital of Nanjing Medical University, Wuxi 214023, China; ^2^Department of Laboratory Diagnosis, Changhai Hospital of the Second Military Medical University, Shanghai 200433, China

## Abstract

Rheumatoid arthritis (RA) is a complex and not fully understood autoimmune disease associated with multijoint damage. The main effector cells, the synovial fibroblasts, are apoptosis resistant and hyperplastic which indicate that autophagy level is high in synovial tissue. Real-time PCR, immunocytochemistry, and western blotting were used in this paper to study the autophagy status of the synovial tissues obtained from RA and OA patients at the time of joint replacement surgery. We further evaluated the correlation between autophagy levels with RA activity-associated serum markers with SPSS. The results showed that the expression levels (both in mRNA and in protein level) of autophagy-related proteins (belcin1, Atg5, and LC3) in the synovial tissue of patients with active rheumatoid arthritis (*n* = 20) were significantly higher than those in OA patients (*n* = 16). We further showed that the LC3-II/*β*-actin relative gray value was strongly correlated with the serum levels of several RA activity-related markers: CRP, ESR, CCP, and RF. Our results indicate that evaluating the autophagy level of synovial biopsies might be a useful way to diagnose RA and to estimate the disease activity. Reducing the expression level of autophagy-related genes might become a new therapeutic target for active rheumatoid arthritis.

## 1. Introduction

Rheumatoid arthritis (RA) is a very common systemic autoimmune disease involving multiple organs, including the lungs, heart, kidneys, eyes, blood vessels, and nerves. The population at the highest risk for developing this disease is women who are 40–60 years old. It seems that multiple pathogenic factors may act synergistically in RA; however, the precise etiology of RA is still unknown. The major pathological feature of RA is angiogenesis in the synovial tissues, which causes synovitis, cartilage destruction, and pannus growth. Because of recent advances in the field, the prevention, diagnosis, and treatment of this disease are entering a new era [1].

Many serious diseases and conditions, such as infection, cancer, neurodegenerative disease, and heart disease are linked closely with autophagy [2–5]. Several regulatory factors that may play key roles in autophagy processes have been discovered in recent years, such as beclin1, which is the key regulatory factor in the autophagy start-up process, and microtubule-associated protein-light chain 3 (LC3) and autophagy-related gene 5 (Atg5), which are components of autophagosomes. In the autophagy process, LC3-I accumulates in autophagosomes and converts to LC3-II. The LC3-II then becomes structural components of the double-membraned autophagosome before being redistributed among the membrane fractions. Therefore, LC3-II can be regarded as a protein marker that reflects the level of autophagy [6–10].

The abnormal apoptosis of rheumatoid arthritis synovial fibroblasts (RASFs) plays a critical role in the progression of RA. It can eventually cause synovial lining hyperplasia and lead to a series of pathological changes [11–13]. Findings from recent in vitro and animal studies have indicated that inhibiting the autophagy of RASFs can increase their apoptosis rate suggesting a potential mutual regulation between apoptosis and autophagy in RASFs [14, 15]. Further exploring the autophagy level in synovial tissue from the injured synovial tissue of patients with active RA and its relationship with serum markers of disease activity will help to provide new proofs of the important roles of autophagy in the progression of RA disease in human being and thus help to disclose new therapeutic means for RA.

In this study, the expression levels of autophagy-related genes and proteins from synovial tissue of RA and osteoarthritis (OA) patients were compared. These data were used to explore the correlation between the autophagy level of synovial tissue and RA activity-associated serum markers.

## 2. Patients and Methods

### 2.1. Patients and Sample Collection

During April 2013–April 2014, 20 patients with active RA and 16 OA patients who had undergone joint replacement surgery in the Changhai Hospital were enrolled and analyzed nonselectively. All RA patients enrolled in this study fulfilled the 1987 American College of Rheumatology (ACR) criteria for classification of RA [16]. Active disease was defined by six or more tender or painful joints (out of 68 joint) and six or more swollen joints (out of 66 joint at both screening and baseline visits, plus either C-reactive protein > 7 mg/L or erythrocyte sedimentation rate above the upper limit of normal).  Table 1 shows the clinical characteristics of these patients. After surgery, a bean-sized sample of synovial tissue from each enrolled patient was quickly put into 1 mL RNA later (Invitrogen, Carlsbad, CA, USA), and these specimens were stored at −80°C for using in later experiments. Synovial tissue paraffin sections from the same patients were provided by the Pathology Department of the Changhai Hospital. This study was approved by the ethics committee of Changhai Hospital, and informed consent was obtained from all the patients enrolled.

## 3. Methods

### 3.1. Total RNA Isolation, Reverse Transcription for cDNA Synthesis, and Real-Time PCR Detection

The synovial tissues from all patients were mechanically crushed, their crushed histiocytic plasms were added to 1 mL TRIzol reagent (Invitrogen, Carlsbad, CA, USA), and the total RNA was extracted by following the manufacturer's protocol. The purity of the resulting RNA was analyzed by using the ratio of optical densities (OD) at 260 nm and 280 nm. A sample OD ratio >1.8 was considered qualified for use in further tests. Prime Script™ RT reverse transcriptase (Takara, Tokyo, Japan) and 500 ng total RNA were used to synthesize the single-stranded cDNA on a PCR machine (Rotor Gene 6000, Corbett Research, Sydney, Australia). To measure the relative amounts of beclin1, LC3, Atg5, and GADHP, the cDNAs were subjected to real-time quantitative PCR on a Light cycler 480 II (Roche, Rotkreuz, Switzerland) according to the manufacturer's protocol. The primers were designed and synthesized by Shanghai Sangon Biological Engineering Technology and Service Company. The sequences of these primers are shown in Table 2. The 20 *μ*L PCR amplification system included 10 *μ*L 2 × SYBR® green I mix (Takara, Tokyo, Japan), 0.5 *μ*L forward primer, 0.5 *μ*L reverse primer, and 1 *μ*L cDNA. Amplification conditions were 95°C for 2 min, followed by 40 cycles of 95°C for 5 s, 60°C for 15 s, and 72°C for 15 s. All samples were measured in triplicate. The relative amounts of beclin1, LC3, and Atg5 were calculated from normalized GADPH levels.

### 3.2. Pathologic Examination of the Synovial Tissue

The synovial tissues obtained from patients with active RA and OA patients at the time of joint replacement surgery were fixed in 10% neutral buffered formalin and embedded in paraffin. Blocks (4 *μ*m-thick) were cut and stained with hematoxylin and eosin (H&E). The diagnoses were confirmed by two seasoned pathologists.

### 3.3. Immunochemistry Staining of Autophagy-Related Proteins

The slides were deparaffinized, and a water-bath heating method was used for antigen retrieval. Endogenous peroxidase activity was blocked by treatment with 3% H_2_O_2_ blocking solution for 15 minutes; then the samples were permeabilized with 3% Triton X-100 for 30 minutes. To block nonspecific binding, 5% normal goat serum (Life Technologies, Grand Island, NY, USA) was used. The slides were incubated with rabbit polyclonal anti-beclin1 antibody (1 : 400, Novus biologicals, Littleton, CO, USA) together with rabbit polyclonal anti-Atg5 antibody (1 : 300, Novus biologicals) and rabbit polyclonal anti-LC3 antibody (1 : 300, Novus biologicals) overnight at 4°C. Rabbit IgG (Life Technologies) diluted with phosphate-buffered saline (PBS)/bovine serum albumin (1 : 400) was used as the negative control. After washing with PBS, the slides were incubated with anti-rabbit and anti-mouse secondary antibodies conjugated to horse radish peroxidase (GTVision™ III Detection System, Mo & Rb, Dako, Glostrup, Denmark) for 30 minutes at room temperature. After being washed with PBS again, the slides were incubated with 3,3′-diaminobenzidine substrate (GTVision III Detection System, Mo & Rb, Dako) in the dark for 10 minutes. Slides were then counterstained with hematoxylin and dehydrated with an ethanol gradient. Finally, the slides were mounted using Vectashield mounting medium (Vector Laboratories, Burlingame, CA, USA). Slides were evaluated using a BX61 microscope (Olympus, Tokyo, Japan). A 13-point scale immunoreactive score system was performed to semiquantitatively evaluate the immunohistochemistry staining results. Immunohistochemical reaction was scored by multiplying the percentage of positive cells (0 = no positive cells, 1 = <10%, 2 = 10–50%, 3 = 51–80%, or 4 = >80% positive cells) by their prevalent degree of staining (0 = negative, 1 = weak, 2 = moderate, or 3 = strong) [17].

### 3.4. Western Blotting

The synovial tissues obtained from patients with active RA and OA patients at the time of joint replacement surgery were homogenized in RIPA Lysis Buffer (Beyotime, China) and protease inhibitor cocktail (Roche, Switzerland). Western blotting was performed using the protocol described by Thermo Fisher Scientific. The total protein was resolved in NuPAGE® Novex 12% Bis-Tris Gel 1.0 mm, 10 Well gels (Thermo Fisher Scientific, USA). Immunoblotting was performed using LC3 and beclin1 antibodies. ECL reagents (Thermo Fisher Scientific, USA) were used to visualize signals.

### 3.5. The Detection of Inflammation and Disease Activity Parameters

Blood samples were collected from the enrolled patients with active RA and OA patients to measure their serum levels of serum C-reactive protein (CRP), rheumatoid factor (RF), and anti-cyclic citrullinated peptide antibodies (CCP) and to measure their erythrocyte sedimentation rate (ESR). Serum CRP and RF levels were measured by the immune rate scatter nephelometry method on a special protein analyzer (IMMAGE800, Beckman, Brea, CA) according to the manufacturer's instructions. The linear range of CRP is 0.1–1152 mg/L with a normal reference value of <7 mg/L. The linear range of RF is 20–800 KIU/L with a normal reference value of <20 KIU/L. SP control 349LPC and 350LPC (Randox, UK) were used as internal quality controls for CRP and RF detection, respectively. The serum CCP levels were measured by enzyme-linked immunosorbent assays (ELISAs) (Kexin Biotech, Shanghai, China). The linear range of CCP is 0–1600 RU/mL with a normal reference value of <25 RU/mL. The ESR was measured by the infrared barrier method on an automatic dynamic sedimentation analyzer (MONITOR 100, Vital Diagnostics, USA) according to the manufacturer's instructions. The normal reference value of ESR is 1–20 mm/h.

### 3.6. Statistical Analyses

Age, CRP, ESR, CCP, RF, and the immunoreactive score of autophagy-related proteins are presented as means with standard deviations, and two independent sample* t*-tests were used to detect differences between groups. The relevance of patient sex was analyzed by Chi-square tests for comparisons between two groups. Bivariate correlation analyses were used to analyze the relationships of CRP, ESR, CCP, or RF with the LC3-II/*β*-actin relative gray value. The correlation coefficient* r* was used to indicate the degree of correlation (0.8–1 = extremely strong, 0.6–0.8 = strong, 0.4–0.6 = moderate, 0.2–0.4 = weak, or 0–0.2 = extremely weak/no correlation). *p* < 0.05 was considered statistically significant. Data analyses were performed using SPSS Statistics 21.0 Software (IBM, New York, USA).

## 4. Results

### 4.1. Clinical Characteristics of Patients with Active RA and OA Patients

A total of 20 patients with active RA and 16 OA patients were enrolled in this study, and a comparative analysis of the clinical features and laboratory data from the two patient groups was carried out (Table 1). Serum CRP and ESR levels were significantly higher in RA patients than in OA patients (*p* < 0.001). The serum levels of CRP in all 20 RA patients were over 8 mg/L, and the ESR levels in 18 out of 20 (90%) RA patients were over 20 mm/h. Both serum levels of CRP and ESR in all 16 OA patients were within the normal reference value.

The positive rate of serum CCP and RF were also significantly higher in RA patients than in OA patients. In RA patients, the positive rate of serum CCP was 90% (18 out of 20 patients), and its concentration range was 25–273.01 RU/mL. Similarly, the positive rate of serum RF in RA patients was 95% (19 out of 20 patients), and its concentration range was 25–143.3 KIU/L. In contrast, the positive rates of serum CCP and RF in OA patients were both 6% (1 out of 16 patients).

### 4.2. The Expression Levels of Autophagy-Related Proteins in the Synovial Tissue from Patients with Active RA Were Significantly Higher Than Those from OA Patients

**Figure 1:**
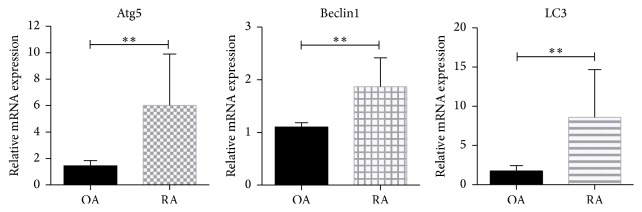
The relative expression levels of beclin1, Atg5, and LC3 mRNA in synovial tissue. The levels of beclin1, Atg5, and LC3 mRNA expression in synovial tissue from RA (*n* = 20) and OA (*n* = 16) patients. ^*∗∗*^*p* < 0.01; the error bars indicate standard deviation.

**Figure 2:**
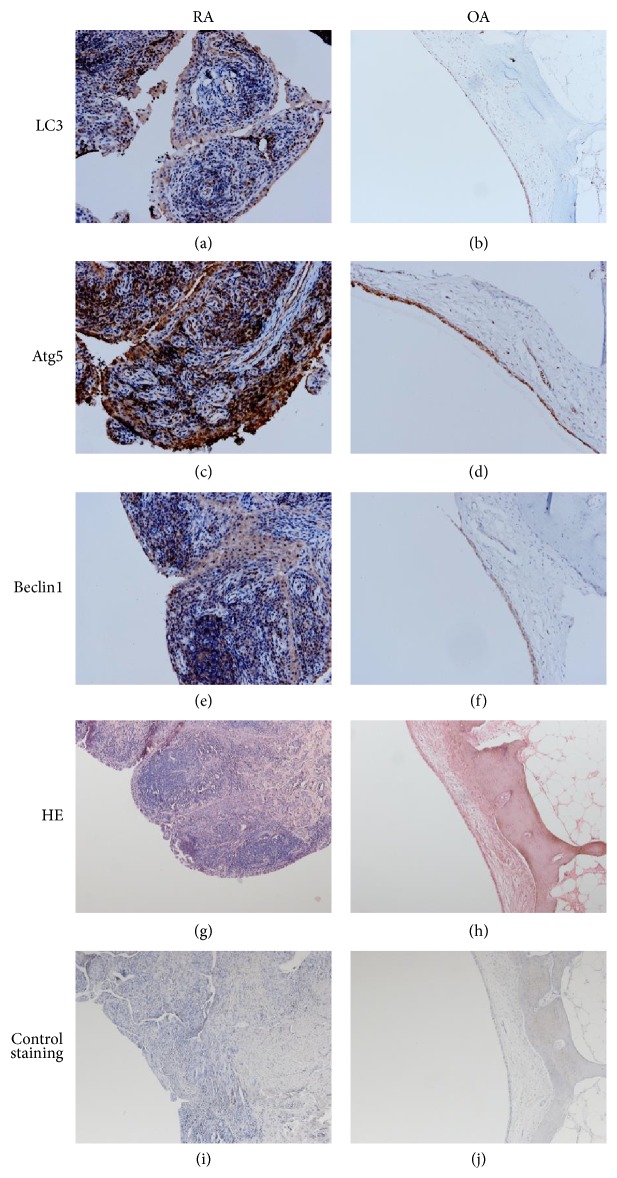
Localization and expression level of beclin1, Atg5, and LC3 in RA synovial tissues. Immunohistochemical analyses of beclin1, Atg5, and LC3 localization in RA synovium. Synovial tissues from RA and OA patients were stained with anti-beclin1 antibodies (a and b), anti-Atg5 antibodies (c and d), anti-LC3 antibodies (e and f), H&E staining (g and h), or anti-rabbit IgG antibodies as a nonimmune control (i and j). Magnification 200x.

To evaluate the differences in autophagy levels between patients with active RA and OA patients, real-time PCR and immunochemistry were applied to detect the expression levels of autophagy-related proteins (beclin1, Atg5, and LC3). The mRNA relative expression levels of beclin1, Atg5, and LC3 in synovial tissue from RA patients were statistically higher than those from OA patients (*p* < 0.001, Figure 1). Based on the immunochemistry results, the protein expression levels of beclin1, Atg5, and LC3 in the synovial tissue from RA patients were also significantly higher than those from OA patients. In synovial tissues from both RA and OA patients, the positively stained cells were mainly located in the synovial membrane, and the positive expression of beclin1, Atg5, or LC3 was indicated by the diffuse cytoplasmic brown-yellow or brown particles located in the cell cytoplasm (Figure 2). Using the 13-point scale immunoreactive score system described in Methods, we analyzed the pathological sections from RA and OA patients for their percentage of positively stained cells and prevalent degree of staining. The immunohistochemical reactions of synovial tissue sections from the RA and OA patients were scored and analyzed (Table 3). The immunohistochemical reaction scores of autophagy-related proteins (beclin1, Atg5, and LC3) were significantly higher in RA patients than in OA patients (*p* < 0.001).

**Figure 3:**
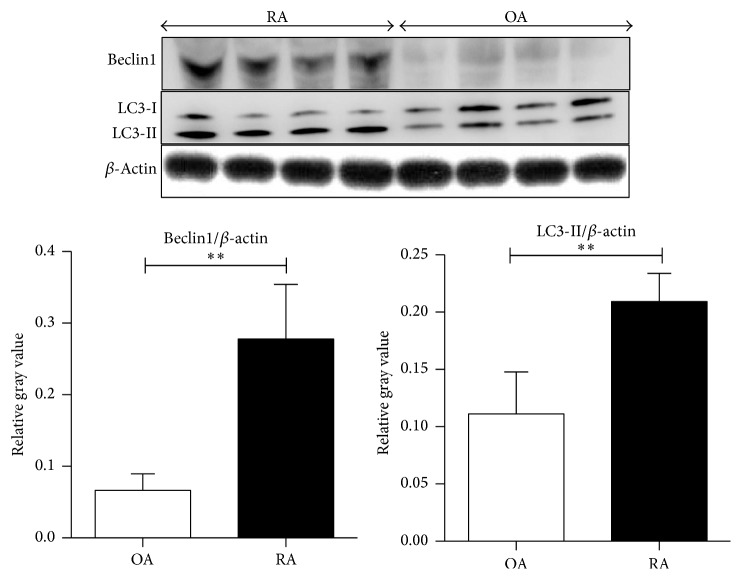
The autophagy-related proteins beclin1 and LC3-II show enhanced expression in RA and OA synovial tissues. Western blotting was used to determine the expression level of autophagy-related proteins beclin1 and LC3-II in RA and OA synovium. The expression of beclin1 and LC3-II was quantified, compared with the expression of actin.

The increase in type II LC3 and beclin1 was confirmed by western blotting analysis with LC3 and beclin1 antibody. The expression levels of LC3-II and beclin1 in synovial tissue from patients with active RA (*n* = 20) were also significantly higher than those from OA patients (*n* = 16) (*p* < 0.001, Figure 3).

### 4.3. The Relationship between Clinical Characteristics and the Autophagy Level of Synovial Tissue

**Figure 4:**
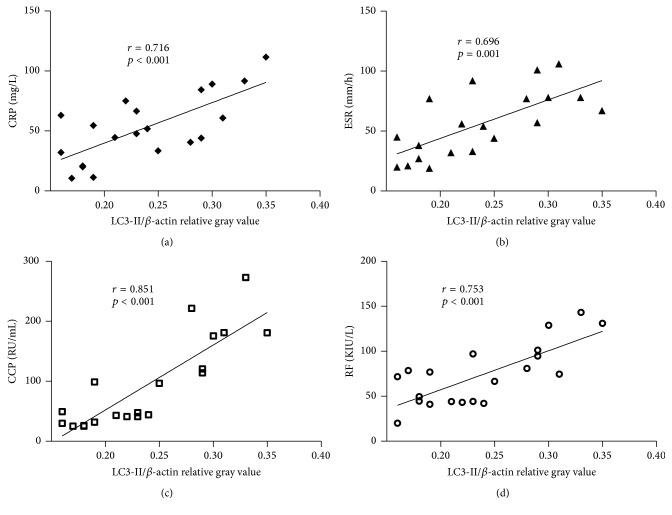
Correlation between the LC3-II/*β*-actin relative gray value and disease activity parameters in RA patients. The correlation between the LC3-II/*β*-actin relative gray value and the serum CRP level (a), ESR level (b), serum CCP level (c), or serum RF level (d). *n* = 20; *p* < 0.05 is considered statistically significant.

LC3-II has been widely applied as an indicative marker for autophagy. Thus, in this study we performed bivariate correlation analyses to analyze the relationship between the LC3-II/*β*-actin relative gray value and the disease activity parameters in patients with active RA. The results show that the LC3-II/*β*-actin relative gray value was strongly correlated with the serum levels of several RA activity-related markers (Figure 4): CRP (*p* < 0.001, *r* = 0.716), ESR (*p* = 0.001, *r* = 0.696), CCP (*p* < 0.001, *r* = 0.851), and RF (*p* < 0.001, *r* = 0.753).

## 5. Discussion

Synovial lining hyperplasia, chronic infection, and autoimmune response are the top three clinical features of RA. The lesions caused by these pathologies could ultimately lead to long-term joint damage resulting in severe joint pain and functional incapacitation. Numerous studies have shown that hyperplasia of the synovial membrane is the most essential factor for causing chronic joint inflammation and destruction of cartilage and bone. RASFs and inflammatory cells play a key role in the pathogenesis of RA. They synthesize and secrete many inflammatory factors, chemokines, and matrix metalloproteinases (MMPs), which can accelerate the process of synovium hyperplasia and pannus formation [18]. Several reports show a reduced amount of apoptosis in the synovial fibroblasts during RA both in vivo (using an experimental arthritis animal model and RA patients [19]) and in vitro (using cultured RASFs [20–22]). The decrease in the RASF apoptosis rate might also contribute to the rapid proliferation of RASFs, and this could ultimately lead to synovial thickening [23, 24]. However, the mechanism for this reduced apoptosis rate has not been fully elucidated. Recent findings demonstrate that there is a very complicated balance mechanism between autophagy and apoptosis in most physiological and pathological processes. Autophagy principally serves an adaptive role to protect cells against nutrient deficiency and other adverse factors, and it plays a crucial role in the pathogenesis and development of a wide variety of illnesses [25, 26]. Previous work has shown that autophagy is involved in the pathological process of joint injury, but most of these studies only conducted in vitro experiments. Very few clinical studies about the autophagy of synovial tissue in RA patients have been conducted, and the clinical data are not clearly stated [27]. Our previous research demonstrated that the inhibition of apoptosis induced by an upregulation of autophagy may participate in the progressive synovial thickening of RA patients [28]. In this study, we discovered significantly increased expression levels of autophagy-related proteins (beclin1, Atg5, and LC3) in the synovial tissue of patients with active RA which indicated that the autophagy pathways within RASFs and other inflammatory cells were significantly activated in the synovial tissue of patients with active RA as compared to that in OA patients. Our results convinced Xu et al.'s [27] findings, and we further evaluated the clinical meaning of the increased autophagy levels in active RA.

Several serological markers are widely used for the diagnosis of RA and evaluation of its disease activity. Previous work suggested that the diagnostic efficiency of RA can be improved by the clinical application of RF and CCP as diagnostic markers. Based on these findings, RF and CCP are currently included in the new classification criteria for RA that was issued by the ACR and the European League Against Rheumatism (ELAR) in 2010 [29]. Recent studies additionally showed that the serum level of CCP, RF, CRP, and ESR was highly relevant to RA disease activity and progression, and it would also be useful for evaluating the prognosis and guiding the treatment of RA [30–34]. Together with RF and CCP, CRP and ESR were included in the classification criteria for RA issued by ACR/ELAR in 2010 [29]. To our knowledge, our work is the first to reveal that the autophagy level of synovial tissue in patient with active RA was positively correlated with disease activity markers. Therefore, our results provide direct experimental evidence for this speculation. For further research, we studied the relationship between the autophagy level of synovial tissue and disease activity markers. With immunochemistry analysis, we showed solid evidences that autophagy-related proteins (beclin1, Atg5, and LC3) were strongly correlated with the serum ESR, CRP, CCP, and RF levels in RA patients. Autophagy is a continuous and dynamic process, and the concept of autophagic flux is important for fully evaluating the autophagy status. We studied the LC3 conversion (LC3 I to LC3-II) with western blotting to indicate the autophagic flux, and unsurprisingly, the LC3-II/*β*-actin was significantly higher in RA patients than in OA patients. Evaluating the autophagy status of synovial tissues by immunohistochemistry through synovial biopsy may help to diagnosis and determine the disease activity in certain patients [35].

The synovial tissues in RA consisted of excessive proliferated synovial cells and all kinds of infiltrated inflammatory cells (lymphocytes, dendritic cells, monocytes, and so on) [1]. Although our study showed evidences that the autophagy level of synovial tissue in patients with active RA was significantly higher than that in OA patients, we do not know the autophagy levels in different cell types. It will take weeks of work to separate different cell types. There is no sense in detecting autophagy status of different cell types cultured in vitro, because in vitro environment cannot equate the microenvironment in vivo according to which autophagy status will change. It is widely accepted that both synovial cells and inflammatory cells play crucial roles in the pathogenesis of RA, and what we want is to evaluate the diagnosis value of autophagy status of synovial tissue in certain complex situations.

## 6. Conclusions

In conclusion, the results of this research indicate that the autophagy level of synovial tissue in patients with active RA was significantly higher than that in OA patients and that the autophagy level was also strongly correlated with the serum ESR, CRP, CCP, and RF levels. Thus, the autophagy level in synovial tissue was significantly correlated with disease activity and severity. This work is clinically valuable because examining the autophagy level of synovial biopsies might be a useful test to diagnose RA and to estimate the disease activity. Reducing the expression level of autophagy-related genes might become a new therapeutic target for RA.

## Figures and Tables

**Table 1:** Clinical data for RA and OA patients.

	RA patients (*n* = 20)	OA patients (*n* = 16)	*p*
Male-to-female ratio	3/17	6/10	*p* > 0.05
Age (mean ± s.d., years)	59.7 ± 11.3	66.9 ± 9.8	*p* > 0.05
CRP (mean ± s.d., mg/L)	52.7 ± 28.0	8.9 ± 6.3	*p* < 0.001
ESR (mean ± s.d., mm/h)	56.1 ± 27.4	9.1 ± 6.0	*p* < 0.001
CCP (mean ± s.d., RU/mL)	93.3 ± 75.5	25.4 ± 1.8	*p* < 0.001
RF (mean ± s.d., KIU/L)	74.0 ± 33.7	20.3 ± 1.3	*p* < 0.001

*RA*, rheumatoid arthritis; *OA*, osteoarthritis; *CRP*, C-reactive protein; *ESR*, erythrocyte sedimentation rate; *CCP*, anti-cyclic citrullinated peptide antibodies; *RF*, rheumatoid factor.

**Table 2:** Primers used for real-time PCR.

Name	Sequence (5′–3′)	Annealing temp (°C)
BECN1	Sense: GGATGGTGTCTCTCGCAGAT	59.8
	Antisense: CAGTCTTCGGCTGAGGTTCT	59.8
LC3	Sense: ACCCTGAGTCTTCTCTTCAGGT	60.2
	Antisense: GTTGCGCTTCACAACTCAGG	60.0
Atg5	Sense: AGGCAACCTGACCAGAAACA	57.8
	Antisense: GAGGAAAGCAGAGGTGATGC	59.8
GADPH	Sense: CAGGAGGCATTGCTGATGAT	58.4
	Antisense: GAAGGCTGGGGCTCATTT	56.3

*BECN1*, beclin1 autophagy-related gene; *LC3*, microtubule-associated protein-light chain 3; *Atg5*, autophagy-related gene 5; *GADPH*, glyceraldehyde-3-phosphate dehydrogenase.

**Table 3:** The immunohistochemical reaction score of RA and OA patients (mean ± s.d.).

	RA patients (*n* = 20)	OA patients (*n* = 16)	*p*
BECN1	8.3 ± 0.9	1.6 ± 0.8	*p* < 0.001
LC3	7.3 ± 1.9	1.2 ± 0.4	*p* < 0.001
Atg5	8.5 ± 0.9	1.7 ± 0.8	*p* < 0.001

*RA*, rheumatoid arthritis; *OA*, osteoarthritis;* BECN1*, beclin1 autophagy related gene; *LC3*, microtubule-associated protein-light chain 3; *Atg5*, autophagy-related gene 5.

## Abbreviations

ACR: American College of Rheumatology
Atg5: Autophagy-related gene 5
CCP: Anti-cyclic citrullinated peptide antibodies
CRP: C-reactive protein
ELISAs: Enzyme-linked immunosorbent assays
ESR: Erythrocyte sedimentation rate
LC3: Microtubule-associated protein-light chain 3
OASF: Osteoarthritis synovial fibroblasts
RA: Rheumatoid arthritis
RASFs: Rheumatoid arthritis synovial fibroblasts.
